# Comparative Proteomic Analysis Provides insight into the Key Proteins as Possible Targets Involved in Aspirin Inhibiting Biofilm Formation of *Staphylococcus xylosus*

**DOI:** 10.3389/fphar.2017.00543

**Published:** 2017-08-21

**Authors:** Chang-Geng Xu, Yan-Bei Yang, Yong-Hui Zhou, Mei-Qi Hao, Yong-Zhi Ren, Xiao-Ting Wang, Jian-Qing Chen, Ishfaq Muhammad, Shuai Wang, Di Liu, Xiu-Bo Li, Yan-Hua Li

**Affiliations:** ^1^College of Veterinary Medicine, Northeast Agricultural University Harbin, China; ^2^Heilongjiang Academy of Agricultural Sciences Harbin, China; ^3^Feed Research Institute, Chinese Academy of Agricultural Sciences Beijing, China; ^4^Heilongjiang Key Laboratory for Animal Disease Control and Pharmaceutical Development Harbin, China

**Keywords:** proteomics, *Staphylococcus xylosus*, biofilm, aspirin, iTRAQ, target

## Abstract

*Staphylococcus xylosus* is an opportunistic pathogen that causes infection in humans and cow mastitis. And *S. xylosus* possesses a strong ability to form biofilms *in vitro*. As biofilm formation facilitates resistance to antimicrobial agents, the discovery of new medicinal properties for classic drugs is highly desired. Aspirin, which is the most common active component of non-steroidal anti-inflammatory compounds, affects the biofilm-forming capacity of various bacterial species. We have found that aspirin effectively inhibits biofilm formation of *S. xylosus* by Crystal violet (CV) staining and scanning electron microscopy analyses. The present study sought to elucidate possible targets of aspirin in suppressing *S. xylosus* biofilm formation. Based on an isobaric tag for relative and absolute quantitation (iTRAQ) fold-change of >1.2 or <0.8 (*P*-value < 0.05), 178 differentially expressed proteins, 111 down-regulated and 67 up-regulated, were identified after application of aspirin to cells at a 1/2 minimal inhibitory concentration. Gene ontology analysis indicated enrichment in metabolic processes for the majority of the differentially expressed proteins. We then used the Kyoto Encyclopedia of Genes and Genomes (KEGG) pathway database to analyze a large number of differentially expressed proteins and identified genes involved in biosynthesis of amino acids pathway, carbon metabolism (pentose phosphate and glycolytic pathways, tricarboxylic acid cycle) and nitrogen metabolism (histidine metabolism). These novel proteins represent candidate targets in aspirin-mediated inhibition of *S. xylosus* biofilm formation at sub-MIC levels. The findings lay the foundation for further studies to identify potential aspirin targets.

## Introduction

*Staphylococcus xylosus* is a gram-positive, coagulase-negative staphylococcus (CNS) with a low G+C content (Planchon et al., 2009). It is naturally present in raw meat or milk and is frequently isolated from subclinical cow mastitis (Planchon et al., 2007; Tremblay et al., 2013). This species is normally regarded as non-pathogenic, however *S. xylosus* has been associated with infections in humans and cow as opportunistic and emerging pathogens (Conrad and West, 1984; Siqueira and Lima, 2002; Pyorala and Taponen, 2009; Akhaddar et al., 2010; Tremblay et al., 2013). In addition, *S. xylosus* exhibits strong ability of biofilm formation (Planchon et al., 2006), which may facilitate its transmission and survival in the environment (Tremblay et al., 2013). Accordingly, biofilm formation complicates the treatment of *S. xylosus* infections.

Biofilms, which are complex three-dimensional structures comprising of cell aggregates encased within a self-produced matrix of extracellular polymeric substances that are adherent to each other and/or a surface (Davey and O’Toole, 2000; Bjarnsholt et al., 2013a; Flemming et al., 2016). And they are particularly problematic in clinical environments, in which bacteria form biofilms *in vivo* and *in vitro* (Cousins et al., 2007). The development of *in vitro* biofilms comprises three stages: (i) attachment, (ii) maturation, and (iii) dispersion (Bjarnsholt et al., 2013b). Biofilm formation is a concerted process controlled by a complex network of regulators that also control metabolism and protein expression. According to previous studies, biofilm formation in staphylococcal species were associated with some factors, such as PIA production (Prasad et al., 2013), changes in amino acid metabolism (Chen et al., 2014), synthesis of exopolysaccharides (EPSs) (Prasad et al., 2013) and metabolic changes (Allan et al., 2014). Compared with other bacterial pathogens, there are some differences about biofilm formation. Such as: *Yersinia pestis*, a deadly bacterial agent, yet only *Y. pestis* forms biofilms in fleas and it is not required for early-phase transmission for biofilm formation (Darby, 2008; Vetter et al., 2010). In addition, the fully mature *Candida* biofilms consisted of a dense network of yeasts, hyphae, and pseudohyphae, and extracellular polymeric material (Ramage et al., 2005). It is generally known, because of biofilm, the antibiotic resistance capability of bacterial strains increase about 10–1000 fold. However, biofilm resistance is a complex multifactorial phenomenon which still remains to be fully elucidated and understood. Different mechanisms may be responsible for the intrinsic resistance.

Biofilms are ubiquitous in nature and notoriously resistant to antimicrobial agents, including biocides, antibiotics, and antiseptics (Gilbert et al., 2002). So, the discovery of new medicinal properties for classic drugs to inhibit *S. xylosus* biofilm formation is highly desired. Aspirin (acetylsalicylic acid), a synthetic compound introduced for treating humans more than 100 years ago (Stepanovic et al., 2004), is a very popular antipyretic, anti-inflammatory, and analgesic that is the most common active component of non-steroidal anti-inflammatory drugs. Additionally, it also affects biofilm formation by various microorganisms (Cabral et al., 2011), including *Candida albicans* (Zhou et al., 2012), *Staphylococcus epidermidis* (Teichberg et al., 1993), *Escherichia coli* (Kang et al., 1998), and *Pseudomonas aeruginosa* (El-Mowafy et al., 2014). However, the study of aspirin inhibiting biofilm formation of *S. xylosus* has not been found.

Many researchers have employed high-throughput proteomic tools to analyze the entire proteome of microorganism as a comprehensive approach to elucidate the major putative targets that are directly or indirectly involved in biofilm formation and to gain specific insights into the physiological and metabolic versatility. Planchon et al. (2009) gained insight into the protein determinants of biofilm formation by *S. xylosus* C2a via comparative proteomic analysis, however these researchers only focused on differential expression between planktonic and sessile cells. In this study, possible targets of aspirin-mediated inhibition of biofilm formation were identified using isobaric tags for relative and absolute quantitation (iTRAQ). And based on our results, which was to lay a foundation for biofilm treatment and identify new potential targets of aspirin.

## Materials and Methods

### Growth of *S. xylosus* Planktonic Cells and Determination of Minimal Inhibitory Concentration Assays of Aspirin

*Staphylococcus xylosus* ATCC 700404 was grown in Tryptic Soy Broth (TSB; Summus Ltd., Harbin, Heilongjiang, China) in 100-mm polystyrene Petri dishes at 37°C for 24 h. Minimal inhibitory concentration (MIC) assays of aspirin were done three times (refer to Yang et al., 2016) with a few modifications. Briefly, *S. xylosus* ATCC700404 was grown aerobically at 37°C in TSB (Summus, Ltd., Harbin, Heilongjiang, China) overnight. The overnight cultures were diluted in sterile physiological saline (corresponding to 1 × 10^8^ colony-forming units/mL). Then, dilute the cultures of *S. xylosus* ATCC700404 1:100 using sterile TSB (Summus, Ltd., Harbin, Heilongjiang, China). Finally, samples (100 μL) were added to the wells of a 96-well plate (Corning Costar^®^3599, Corning, NY, United States) containing serial dilutions of aspirin in culture medium. Control bacteria were cultivated in the absence of aspirin. The MIC was determined as the lowest concentration of aspirin that completely inhibited *S. xylosus* growth after incubation for 24 h at 37°C.

### TCP Assay for Determining the Effects of Aspirin on Biofilm Formation

TCP assay for determining the effects of biofilm formation was carried out as described previously (Wang et al., 2016). Briefly, *S. xylosus* ATCC 700404 was grown in Tryptic Soy Broth, and 100-μL was added to each well of a 96-well microplate containing an equal volume of aspirin solution to achieve final concentrations of 1/2 (0.625 mg/mL), 1/4 (0.3125 mg/mL), 1/8 (0.15625 mg/mL), and 1/16 (0.078125 mg/mL) MIC. A negative control (TSB alone) and a positive control (bacteria alone) were also included. After incubation at 37°C for 24 h without shaking, all wells were then washed with sterile PBS and stained with crystal violet indicator. The amount of released stain was quantified by measuring the absorbance at 595 nm using a microplate reader. The reported values are the means of three measurements. The experiments were performed in triplicate.

### Determination of the Growth-Inhibiting Activity of Aspirin

The growth rates of *S. xylosus* ATCC 700404 treated with aspirin at 1/2, 1/4, 1/8, and 1/16 MIC doses and untreated cells were analyzed (Yang et al., 2015). Briefly, cells treated without and with aspirin (1/2, 1/4, 1/8, and 1/16 MIC) were incubated at 37°C for 24 h and sampled every hour to measure the OD_600_.

### Scanning Electron Microscopy (SEM) Observation

The procedure of Yang et al. (2016) was followed. Briefly, cultures were diluted to an optical density of 0.1 at 600 nm (OD_600_) and 2 mL was added to wells of a 6-well microplate containing an 10 mm × 10 mm sterilized rough organic membrane (Mosutech, Co., Ltd., Shanghai, China) respectively on the bottom. After incubation without shaking for 24 h at 37°C, medium and planktonic bacteria on the organic membrane were removed with sterile PBS. The biofilms obtained from bacterial cells and prepared for analysis as described by Yang et al. (2016).

### Preparation of Protein Extracts

*Staphylococcus xylosus* ATCC 700404 was grown in TSB (Summus, Ltd., Harbin, Heilongjiang, China) in 100-mm polystyrene Petri dishes at 37°C for 24 h. The supernatants were removed, and the dishes were washed twice with Tris-HCl buffer (50 mM, pH 7.5). The biofilms were detached by scraping. After sonication for 5 min (Bransonic 220; Branson Consolidated Ultrasonic Pvt Ltd., Australia), the cells were centrifuged at 12,000 ×*g* for 10 min at 4°C. The cell pellets were washed twice with Tris-HCl buffer. *S. xylosus* was also grown in TSB containing 1/2 MIC (0.625 mg/mL) aspirin in 100-mm polystyrene Petri dishes, as described above. The experiments were performed in triplicate.

### Protein Digestion and iTRAQ Labeling

Protein digestion was performed according to the filter-aided sample prep (FASP) procedure described by Zhao et al. (2015). iTRAQ sample labeling was performed using an iTRAQ Reagent-8plex Multiplex Kit (AB Sciex U.K. Limited) according to the manufacturer’s instructions. Briefly, 200 μg of protein under two different conditions (cells treated with aspirin at 1/2 MIC and untreated cells) for each sample was added to 30 μL STD buffer [4% sodium dodecyl sulfate (SDS), 100 mM dithiothreitol (DTT), 150 mM Tris-HCl, pH 8.0]. The detergent, DTT and other low-molecular-weight components were removed using UA buffer (8 M urea, 150 mM Tris-HCl, pH 8.0) via repeated ultrafiltration (Microcon filter units, 30 kDa). Then, 100 μL 0.05 M iodoacetamide in UA buffer was added to block reduced cysteine residues, and the samples were incubated for 20 min in the dark. The filters were washed three times with 100 μL UA buffer and then twice with 100 μL DS buffer (50 mM triethyl ammonium bicarbonate at pH 8.5). The protein suspensions were digested with 2 μg of trypsin (Promega) in 40 μL of DS buffer overnight at 37°C, and the resulting peptides were collected as filtrates. The peptide contents were estimated by performing ultraviolet (UV) light spectral density measurements at 280 nm using an extinction coefficient of 1.1 with a 0.1% (g/L) solution that was calculated based on the frequency of tryptophan and tyrosine in vertebrate proteins. For labeling, each iTRAQ reagent was dissolved in 70 μL ethanol and added to the peptide mixture.

The peptides from *S. xylosus* biofilms treated with aspirin and untreated samples were labeled with 113 and 115 isobaric reagents, respectively. The samples were multiplexed and vacuum-dried. Three independent biological experiments were performed.

### Peptide Fractionation with Strong Cation Exchange (SCX) Chromatography

The iTRAQ-labeled peptides were fractionated by strong cation exchange (SCX) chromatography using the AKTA Purifier System (GE Healthcare). The dried peptide mixtures were reconstituted and acidified in 2 mL buffer A (10 mM KH_2_PO_4_ in 25% ACN, pH 2.7) and loaded onto a polysulfoethyl 4.6 mm × 100 mm column (5 μm, 200 Å, PolyLC, Inc., Columbia, MD, United States). The peptides were eluted at a flow rate of 1 mL/min with a gradient of 0–10% buffer B (500 mM KCl, 10 mM KH_2_PO_4_ in 25% ACN, pH 2.7) for 2 min, 10–20% buffer B for 25 min, 20–45% buffer B for 5 min and 50–100% buffer B for 5 min. Elution was monitored at 214 nm, and fractions were collected every min. The collected fractions (approximately 30 fractions) were combined into 10 pools and desalted using C18 cartridges [Empore^TM^ SPE Cartridges C18 (standard density), bed I.D. 7 mm, volume 3 mL, Sigma]. Each fraction was concentrated by vacuum centrifugation and reconstituted in 40 μL 0.1% (v/v) trifluoroacetic acid. All samples were stored at -80°C until liquid chromatography–tandem mass spectrometry (LC–MS/MS) analysis was performed.

### LC-Electrospray Ionization (ESI) MS/MS Analysis Using Q Exactive

Experiments were performed using a Q Exactive mass spectrometer coupled to an Easy nLC (Proxeon Biosystems, now Thermo Fisher Scientific). A 10-μL sample of each fraction was injected onto the nano LC–MS/MS for analysis. The peptide mixtures (5 μg) were loaded onto a C18-reversed phase column (Thermo Scientific Easy Column, length: 10 cm, 75-μm inner diameter, 3 μm resin) in buffer A (0.1% formic acid) and separated using a linear gradient of buffer B (80% acetonitrile and 0.1% formic acid) at a flow rate of 250 nL/min over 140 min, controlled by IntelliFlow technology. MS data were acquired using a data-dependent top-10 method that dynamically chose the most abundant precursor ions from the survey scan (300–1800 m/z) for higher-energy collisional dissociation (HCD) fragmentation. Determination of the target value was based on predictive Automatic Gain Control (pAGC). The dynamic exclusion duration was 60 s. Survey scans were acquired at a resolution of 70,000 at m/z 200, and the resolution for HCD spectra was set to 17,500 at m/z 200. The normalized collision energy was 30 eV, and the underfill ratio, which specifies the minimum percentage of the target value likely to be reached at maximum fill time, was defined as 0.1%. The instrument was operated with peptide recognition mode enabled.

### Sequence Database Searching and Data Analysis

MS/MS spectra were searched using the MASCOT engine (Matrix Science, London, United Kingdom; version 2.2) embedded in Proteome Discoverer 1.3 (Thermo Electron, San Jose, CA, United States) against the UniProt *S. xylosus* database (133,549 sequences, downloaded March 3, 2015) and a decoy database. For protein identification, the following options were used: peptide mass tolerance = 20 ppm; MS/MS tolerance = 0.1 Da; enzyme = trypsin, missed cleavage = 2; fixed modification, carbamidomethyl (C), iTRAQ8plex (K), iTRAQ8plex (N-term); variable modification, oxidation (M). Quantification was performed based on the peak intensities of reporter ions in the MS/MS spectra. The ratio of label 113 to 115 represents expression of proteins with protein identification confidence at a 1% false discovery rate (FDR) (Unwin et al., 2010). A minimum of one unique peptide was required to identify a protein; FDR was set to <0.01 to identify both peptides and proteins. Protein quantification was based on the total intensity of the assigned peptides. Proteins were considered overexpressed when the iTRAQ ratio was above 1.2 and underexpressed when the iTRAQ ratio was lower than 0.8 in untreated cells compared to aspirin-treated cells. Protein expression was evaluated by performing paired *t*-tests, and statistical significance was achieved at *P*-values < 0.05.

### Bioinformatics

Sequence data for selected differentially expressed proteins were examined using AgriGO for gene ontology (GO) annotation. The GO project groups protein function into three domains: biological processes, cellular components, and molecular functions. Following annotation and annotation augmentation, enzyme codes were sequentially mapped to annotated sequences and metabolic pathways in the Kyoto Encyclopedia of Genes and Genomes (KEGG). The protein–protein network of significantly differentially expressed proteins was analyzed by STRING software^1^.

### Statistical Analysis

In this study, Student’s *t*-tests were performed to examine deviations between different samples, and *P* < 0.05 indicated a significant difference. Assays were performed three times, and the mean ± standard deviation was computed.

### Results

#### Effects of Aspirin on Biofilm Formation *In Vitro* by the TCP Assay

**FIGURE 1:**
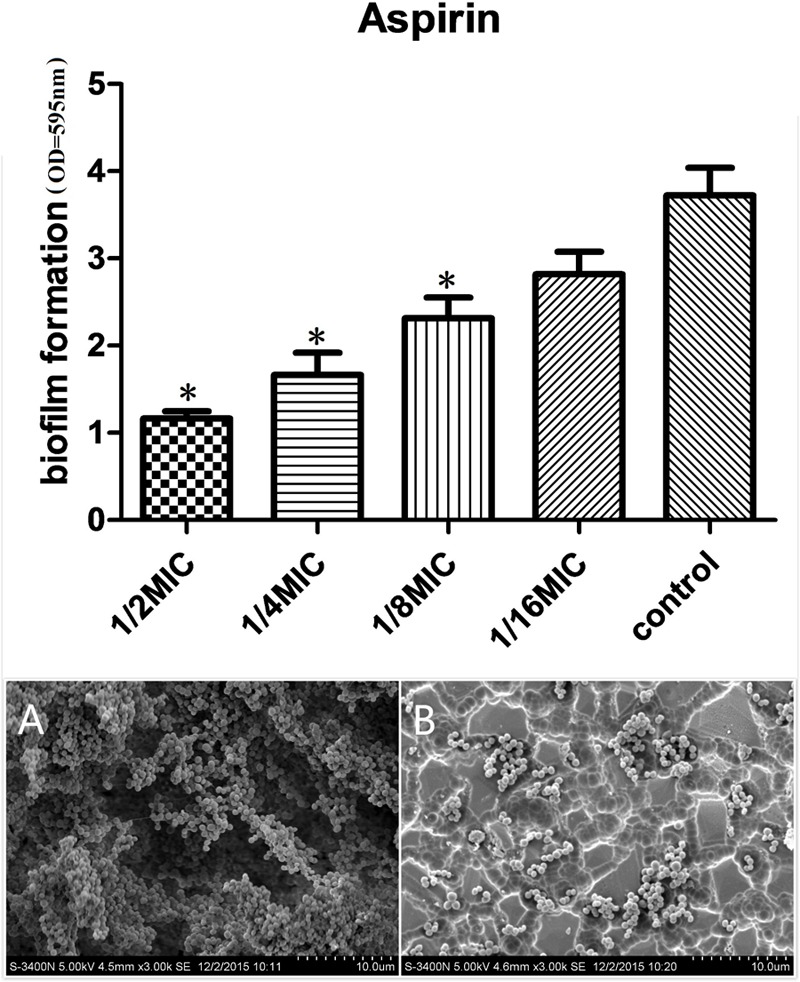
Effects of different concentrations of aspirin on *Staphylococcus xylosus* ATCC 700404 biofilm formation. **(A)** Biofilm formation by *S. xylosus* in the absence of aspirin. **(B)** Biofilm formation by *S. xylosus* in the presence of aspirin at 1/2 MIC. Data are expressed as the mean ± standard deviation. A significant decrease (^∗^*P* < 0.05) was observed compared with control biofilm formation by *S. xylosus in vitro.*

We evaluated the actions of aspirin on biofilm growth *in vitro*. The aspirin MIC against *S. xylosus* was 1.25 mg/mL, and doses at 1/2, 1/4, and 1/8 MIC caused significantly higher reductions in the biofilm-forming abilities of *S. xylosus* compared with the positive control (*P* < 0.05). However, there were no pronounced effects on *S. xylosus* biofilm formation at a 1/16 MIC dose (*P* > 0.05) (**Figure 1**).

### Bacterial Growth under the Influence of Aspirin at Different Sub-MICs

**FIGURE 2:**
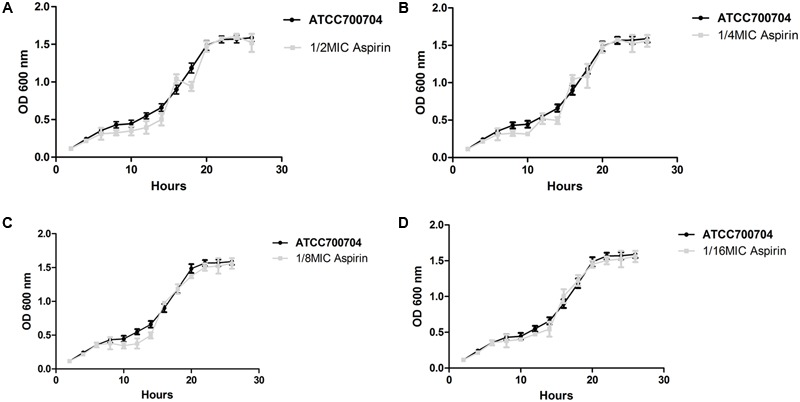
Growth curve of *S. xylosus* ATCC700404 in the absence of aspirin and in the presence of aspirin at doses of, 1/2 **(A)**, 1/4 **(B)**, 1/8 **(C)**, or 1/16 MIC **(D)**. Data are expressed as the mean ± standard deviation.

The viability of *S. xylosus* treated with sub-MIC aspirin was similar to the viability of untreated *S. xylosus*. Both treated and untreated cells reached the stationary phase after 20 h of incubation at 37°C (**Figure 2**), suggesting no effects on the growth rate.

### Direct Observation of Biofilm Formation *In Vitro* by SEM

Scanning electron microscopy (SEM) analysis was performed to observe biofilm formation by *S. xylosus* treated with 1/2 MIC aspirin and untreated *S. xylosus* under the same growth conditions. As shown in **Figure 1A**, in the absence of aspirin, the surface of the glass slide was almost entirely covered by *S. xylosus* aggregates and microcolonies. However, when the culture medium contained aspirin at 1/2 MIC, biofilms were characterized by the presence of small clusters of cells interspersed amongst individual cells (**Figure 1B**). Thus, *S. xylosus* biofilm formation was inhibited by 1/2 MIC aspirin *in vitro.*

### Identification of Differentially Expressed Proteins Using iTRAQ Labeling

Because aspirin inhibits biofilm formation, we performed proteome analysis to obtain further information about proteins down- and up-regulated in the presence of aspirin. To identify differentially expressed proteins, biofilms treated with aspirin or untreated were labeled with isobaric reagents; the samples were pooled, fractionated by SCX chromatography, separated by LC and analyzed by MS/MS. Based on a fold-change of >1.2 or <0.8 (*P*-value < 0.05), 1,762 proteins were identified. We screened 178 differentially expressed proteins following biofilm formation under 1/2 MIC aspirin, of which 111 and 67 proteins were down- and up-regulated, respectively. Detailed information can be found in **Supplementary Table S1**. Meanwhile, raw data can be found in **Supplementary Tables S2, S3, S4**. In order to verify the accuracy of the data, the selected differential proteins were validated at the mRNA level by qPCR analysis, see Supplementary Figure S1. Given the inhibitory effects of aspirin on *S. xylosus* biofilms, special attention was given to proteins that demonstrated significant down-regulation of biofilm proteome in the presence of aspirin.

### GO Analysis and Annotation

**FIGURE 3:**
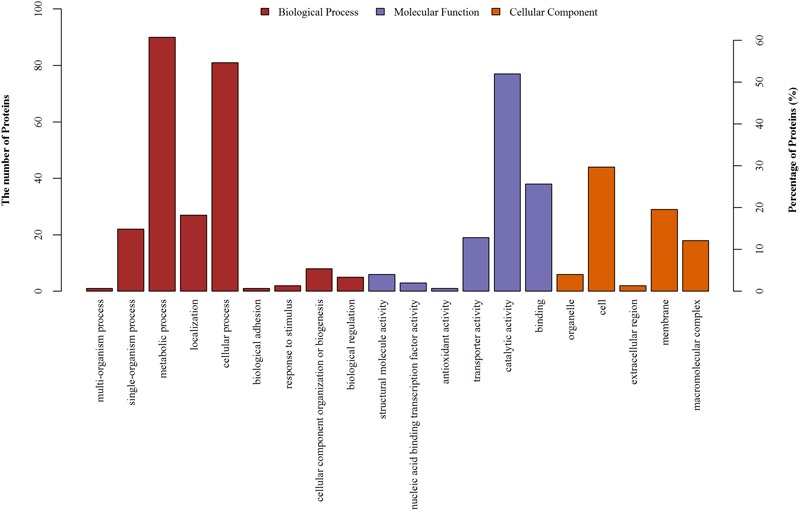
GO annotation of differentially regulated protein (ratio: > 1.2 or < 0.8) functions. Y-axis represented the number of identified proteins in each GO category.

Changes in the expression levels of certain intracellular proteins were observed following aspirin treatment. We performed GO functional annotation for all identified differential proteins. GO is dynamically updated, and its controlled vocabulary describes genes and gene product characteristics. The results comprise a large range of molecular functions, biological processes, and cellular components. A protein may be involved in a number of biological processes, with a variety of molecular functions, and be present in a number of cellular components. There are 20 important functional groups defined by GO analysis, and all identified differential proteins underwent simultaneous category annotation. The majority of differentially expressed proteins found are enriched in metabolic pathways based on analysis of biological processes, followed by cellular processes, single-organism processes, and localization. Molecular functions, which describe the actions of a gene product at the molecular level, included protein functions, such as catalysis or binding. In the cellular component category, proteins associated with macromolecular complexes and the membrane constituted the largest categories. Detailed information is provided in **Figure 3**. We also identified differentially expressed proteins involved in metabolic processes.

### KEGG Pathway Analysis

**FIGURE 4:**
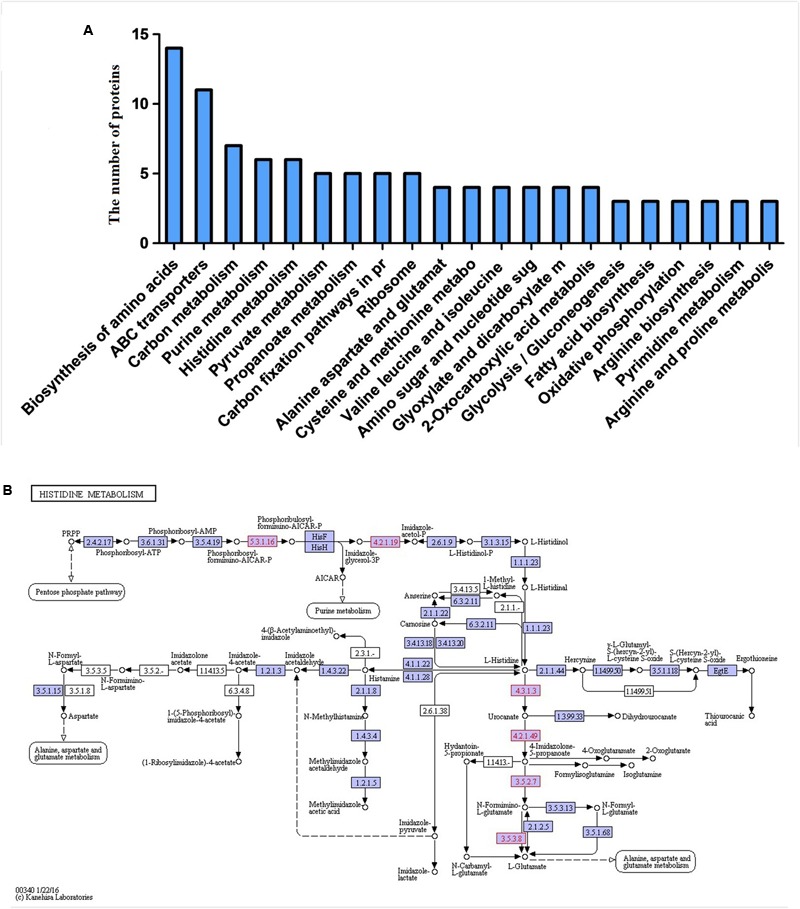
**(A)** Proteins involved in KEGG pathways. **(B)** Protein expression profiles during biofilm formation following treatment with aspirin mapped onto the histidine metabolism pathway. Changes in enzymes (proteins) are marked in green and mapped onto KEGG pathways.

The KEGG database is a collection of manually drawn pathway maps representing the current knowledge of molecular interactions and reaction networks. Molecules are represented as nodes, and the biological relationship between two nodes is represented as an edge (line) (Kanehisa et al., 2012). Pathway annotation to identify differential proteins helps to reveal which proteins may be involved in a metabolic or signaling pathway, and pathway analysis provides a comprehensive, systematic, and direct understanding of cell biology, disease mechanisms, and drug mechanisms of action. We annotated all identified differentially expressed proteins using the KEGG database, with all mapping onto 71 KEGG pathways. Twenty-one pathways were considered statistically significant, including biosynthesis of amino acids, ATP-binding cassette (ABC) transporters, carbon metabolism, and histidine metabolism. Detailed information is presented in **Figure 4A**. Biosynthesis of amino acids included the greatest number of proteins, followed by ABC transporters and carbon and other metabolic pathways. Thus, aspirin may inhibit biofilm formation by interfering with amino acid biosynthesis, transporters, and certain metabolic pathways. Of particular interest, GO analysis revealed significant changes in differentially expressed proteins involved in metabolic processes. Given these results, we then focused on analyzing differentially expressed proteins and metabolic pathways.

### Protein–Protein Interaction Analysis

**FIGURE 5:**
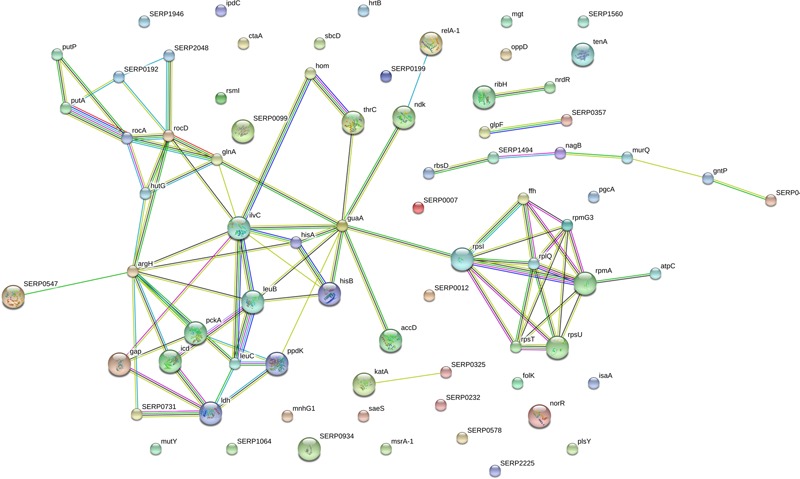
The network of significantly differentially expressed proteins (ratio: > 1.2 or < 0.8 fold) was analyzed by String. Small nodes represent protein of unknown 3D structure large; nodes represent some 3D structure is known or predicted. Colored nodes represent query proteins and first shell of interactors; white nodes represent second shell of interactors. The blue lines represent database evidence; the purple lines represent experimental evidence; yellow lines represent text mining evidence; the black lines represent coexpression evidence; and green lines represent neighborhood evidence.

A network was constituted by protein–protein interaction of the 178 significantly differentially expressed proteins in **Figure 5**. As shown in **Figure 5**, a group of significant differentially expressed proteins were found to be actively interacted including: glutaminesynthetase (glnA), Glyceraldehyde-3-phosphatedehydrogenase (gap), phosphor-glucomutase (pgcA), isocitrate-dehydrogenase (icd), ketol-acid-reductoisomerase (ilvC), imidazoleglycerol-phosphate-dehydratase (HisB), 1-(5-phosphoribosyl)-5-[(5-phosphoribosylamino)methylideneamino]imidazole-4-carboxamide (HisA), there-isopropylmalate dehydrogenase (leuB). So, it is predicted that differentially proteins maybe involved in the forming of biofilm and these novel proteins represent candidate targets in aspirin-mediated inhibition of *S. xylosus* biofilm formation at sub-MIC levels.

## Discussion

In recent years, with the deepening of proteomics research, the relationship between proteomics and pharmaceutical research is becoming more and more closely. Application of iTRAQ (isobaric tags for relative and absolute quantification) can be used to high-throughput analysis of protein expression profiling of drugs treated before and after, so as to discover and confirm the targets of drug. iTRAQ technology has become a very important technique in quantitative proteomics, which has been successfully verified in many organisms and bacteria, such as: biofilm formation of bacterias. Biofilm formation is a bacterial process that facilitates resistance to antimicrobial treatment, necessitating the discovery and development of new antimicrobial therapeutic agents (Taha et al., 2006). Aspirin, an active component of analgesics and non-steroidal anti-inflammatory drugs, induces a number of morphological and physiological alterations in bacteria (Price et al., 2000; Zhou et al., 2012). At the same time, aspirin also can inhibit biofilm formation by *C. albicans* (Zhou et al., 2012), *S. epidermidis* (Teichberg et al., 1993), *E. coli* (Kang et al., 1998), and *P. aeruginosa* (El-Mowafy et al., 2014). Additional studies found that aspirin also suppresses the biofilm formation of *C. guilliermondii, C. kefyr, C. glabrata*, and *C. parapsilosis* (Stepanovic et al., 2004). It has been reported that salicylate can inhibit the production of extracellular polymers (Muller et al., 1998). And quorum-sensing (QS) is believe to influence biofilm formation (Xu et al., 2015), and aspirin acts as a QS inhibitor to effectively inhibit biofilm formation without killing cells in *P. aeruginosa* (El-Mowafy et al., 2014). However, the mechanism underlying the aspirin-mediated inhibition of biofilm formation is complex and unclear. In this study, we focused on identifying possible protein targets, particularly down-regulated proteins, of aspirin-mediated inhibition of *S. xylosus* biofilm formation.

In addition to protein components, amino acids act as signaling molecules and regulators of gene expression. Meanwhile, changes in amino acid metabolism relate to biofilm formation (Chen et al., 2014). L-histidine is one of the 21 proteinogenic amino acids and an essential nutrient for animals but is synthesized by plants and microorganisms (Dietl et al., 2016). Indeed, our proteomic analysis identified novel proteins involved in a previously undescribed histidine metabolism pathway in *S. xylosus.* According to our results, proteins that are directly or indirectly related to histidine metabolism and biosynthesis, such as imidazoleglycerol-phosphate-dehydratase (4.2.1.19),1-(5-phosphoribosyl)-5-[(5-phosphoribosylamino)methylideneamino]imidazole-4-carboxamide (5.3.1.16), histidine ammonia-lyase (4.3.1.3), urocanate hydratase (4.2.1.49) and imidazolonepropionase (3.5.2.7), were clearly down-regulated in the presence of aspirin (**Figure 4B**). 1-(5-phosphoribosyl)-5-[(5-phosphoribosylamino)methylideneamino] imidazole-4- carboxamide (HisA) and imidazoleglycerol-phosphate-dehydratase (HisB) is involved in steps 4 and 6 of the subpathway that synthesizes L-histidine from 5-phospho-alpha-D-ribose 1-diphosphate, respectively. The first enzyme exclusively dedicated to histidine biosynthesis is imidazoleglycerol-phosphate dehydratase (HisB) (Dietl et al., 2016). Histidine ammonia-lyase (hutH) is involved in step 1 of the sub-pathway that synthesizes L-glutamate from L-histidine, and imidazolonepropionase (hutI) is involved in step 3 of the sub-pathway that synthesizes L-glutamate from L-histidine. According to a recent study, L-histidine dramatically decreases biofilm formation of *Saccharomyces cerevisiae Flor yeasts* (Zeidan et al., 2015). In addition, imidazolonepropionase plays a crucial role in histidine degradation in mammals and bacteria (Yang et al., 2008). Interesting, imidazoleglycerol-phosphate-dehydratase (HisB) is conserved among bacteria, lower eukaryotes and plants, but is absent in mammals. This feature makes it an attractive target for herbicide discovery (Ahangar et al., 2013). Deletion of hisB, was also shown to cause histidine auxotrophy (Ahangar et al., 2013). These reports indicate that HisB is a druggable target. Of note, urocanate hydratase (hutU), which catalyzes the synthesis of urocanase, was found to be down-regulated in the presence of aspirin. Urocanase plays a crucial role in the L-histidine degradation pathway, and compared to wild-type *A. baumannii*, a urocanase mutant strain (Δhut) exhibits reduced biofilm (Cabral et al., 2011).

Planchon et al. (2009) observed undetectable levels of glutamine synthetase in *S. xylosus* C2a planktonic cells compared to those in biofilm. However, we observed down-regulation of glutamine synthetase upon aspirin treatment. Increased glutamine potentially promotes biofilm formation by *Bacillus subtilis* (Liu et al., 2015), and in addition to its function in biosynthesis, glutamine synthetase plays a crucial role in pathogenesis and affects the formation of bacterial biofilms. Indeed, genetic disruption of glutamine synthetase leads to a decrease in biofilm formation by *Mycobacterium bovis* (Chandra et al., 2010; Tripathi et al., 2013). These results agree with those obtained in our study and that suggest glutamine synthetase may be a drug target as well as a vaccine candidate. Thus, aspirin may act as glutamine synthetase inhibitor to inhibit biofilm formation.

Glyceraldehyde-3-phosphate dehydrogenase (GAPDH), an enzyme in the glycolytic pathway, is responsible for phosphorylating glyceraldehyde-3-phosphate and participates in membrane fusion, microtubule binding, phosphotransferase activity and repair apoptosis (Oliveira et al., 2012). GAPDH is up-regulated in biofilms formed by various bacterial species (Sauer et al., 2002; Planchon et al., 2009; Wang et al., 2012). Moreover, GAPDH-knockout *S. suis* mutants demonstrate a decreased ability to form biofilms (Wang et al., 2012). Thus, through GAPDH down-regulation, aspirin appears to cause reductions in biofilm formation. Interestingly, phosphoglucomutase (PGM), which is involved in both the pentose phosphate and glycolytic pathways, is primarily responsible for converting glucose 1-phosphate to glucose 6-phosphate and is up-regulated in *S. xylosus* C2a compared to planktonic cells (Planchon et al., 2009). However, based on our results, PGM expression was down-regulated in cells treated with sub-MIC aspirin compared with untreated cells. In several bacterial species, PGM is bifunctional and required for the formation of various EPSs, which play significant roles in the synthesis of active core biofilm compounds and biofilm formation (Levander and Radstrom, 2001; Sahu et al., 2014). Specifically, the PGM enzyme is a potential drug target during biofilm-associated *A. baumannii* infections, as revealed by 3D structural simulation studies (Sahu et al., 2014).

Ketol-acid-reductoisomerase (KARI, ilvC) and 3-isopropylmalate dehydrogenase (leuB) in the same pathway were down-regulated, involved in biosynthesis of amino acids pathway. KARI is a bifunctional enzyme that catalyzes the second and third reactions of the Branched-chain amino acids (BCAA) pathway, converting either 2-acetolactate or 2-aceto-2-hydroxybutyrate to their corresponding 2, 3-dihydr-oxy-3-alkylbutyrate products. Three-isopropylmalate dehydrogenase (IPMD) encoded by LEUB is a key enzyme in leucine (Leu) biosynthetic pathway. Branched-chain amino acids (BCAAs) including leucine (Leu), valine (Val) and isoleucine (Ile), and the biosynthetic pathway for the BCAAs is present in plants, fungi and bacteria, but not in animals, making it an attractive target for herbicidal and antimicrobial drug discovery (Kohlhaw, 2003; McCourt and Duggleby, 2006). Given the success of acetohydroxyacid synthase (AHAS) as a biocide target, other enzymes such as KARI and leuB in the BCAA pathway may also possess great potential as drug targets (Lv et al., 2016). Recent studies have revealed that BCAAs, especially Leu, not only serve as fundamental substrates for protein synthesis but also have unique ability to initiate signal transduction pathways that modulate translation initiation (Yoshizawa, 2004; Kimball and Jefferson, 2006). But it is hard to hypothesize the function of those proteins on the biofilm formation in *S. xylosus* cells. Further works should be done.

**FIGURE 6:**
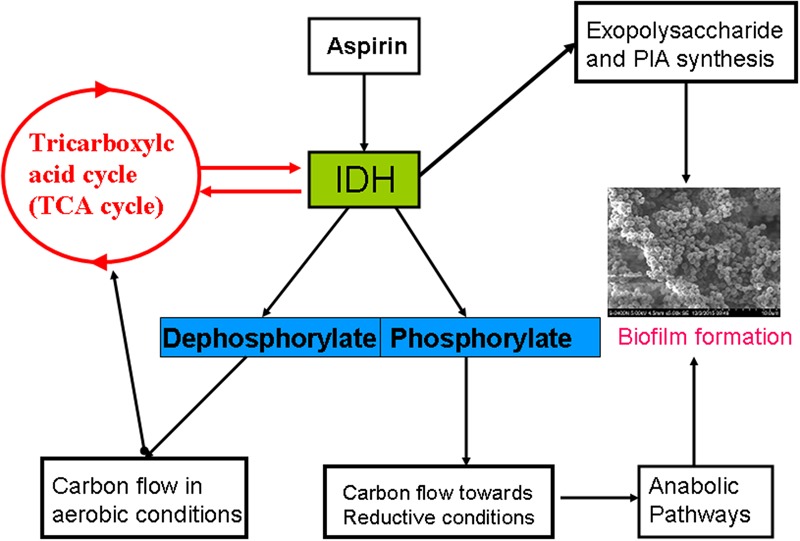
Schematic representation of the effects of aspirin on IDH during biofilm formation.

The TCA cycle is an important metabolic pathway providing significant energy to a cell and is largely regulated by isocitrate dehydrogenase (IDH) expression (Steen et al., 2001). Our finding showed that IDH was down-regulated in aspirin-treated cells. It is consistent with the studies described with Planchon et al. (2009) and Li et al. (2015). TCA cycle repression via an altered IDH metabolic status in *S. aureus* results in massive redirection toward synthesis of EPS and PIA, and ultimately leads to biofilm formation (Prasad et al., 2013). Moreover, in *S. aureus*, IDH is regulated by phosphorylation/dephosphorylation, the most conserved processes involved in intercellular and intracellular communication in both prokaryotes and eukaryotes (Fischer and Krebs, 1989). For example, shifts in phosphorylation/dephosphorylation play vital roles in maintaining redox status in *S. aureus*, which in turn has profound effects on biofilm formation. According to a previous study (Prasad et al., 2015), IDH activity is closely related to biofilm formation. Furthermore, our results also indicated an aspirin-mediated decrease in biofilm formation and the clear down-regulation of proteins that are directly or indirectly related to IDH activity (**Figure 6**).

## Conclusion

This study demonstrates the ability of aspirin to inhibit biofilm formation without killing cells by *S. xylosus* ATCC 700404. Specifically, iTRAQ revealed that aspirin caused down-regulation of various proteins that are directly or indirectly related to biofilm formation. Our data provide information regarding protein expression during *S. xylosus* ATCC 700404 biofilm formation following treatment with sub-MIC doses of aspirin and lay the foundation for identifying novel protein targets of aspirin. In summary, this study demonstrate that aspirin as representative of new medicinal value of the old classic drug can inhibit biofilm formation of *S. xylosus* ATCC 700404 and paves the way for searching for specific targets of biofilm formation.

## Author Contributions

Y-HL designed the whole experiment; C-GX directed the completion of the experiment; Y-BY, Y-HZ, M-QH, Y-ZR, X-TW, J-QC, IM, SW, DL, and X-BL provided help during the experiment.

## Conflict of Interest Statement

The authors declare that the research was conducted in the absence of any commercial or financial relationships that could be construed as a potential conflict of interest.

## Footnotes

1 http://string-db.org/

## Supplementary Material

TABLE S1 List of differentially expressed proteins in aspirin-treated cells.

TABLE S2 Identification of peptide.

TABLE S3 Identification of proteins.

TABLE S4 Total number of proteins in aspirin-treated cells.
