# Supplementary material for: Comparative Proteomic Analysis Provides insight into the Key Proteins as Possible Targets Involved in Aspirin Inhibiting Biofilm Formation of Staphylococcus xylosus
Source: Front Pharmacol. 2017 Aug 21;8:543. doi: 10.3389/fphar.2017.00543 (PMC5566577; doi:10.3389/fphar.2017.00543)
Supplement: Supplementary file 5 [file Table_5.DOC]

**1 Validation of proteomics analysis**

**RNA isolation and Real-time PCR analysis**

Real-time PCR analysis was described in our previous study .To investigate the effect of 1/2MIC of aspirin on expression of genes, *Staphylococcus xylosus* media culture (mid-log growth phase) was supplemented with 1/2 MIC of aspirin and incubated again at 37°C for 24 h. Cells without aspirin served as control. Supplemented solution was centrifuged at 10,000xg for 5 min and afterwards treated with an RNASE REMOVER I (Huayueyang Ltd, Beijing, China). An E.Z.N.A. ™ Bacterial RNA isolating kit was used to determine total RNA levels. Real-time PCR analysis for each sample was carried out as previously described by Yang . The primers of the target genes are listed in Table 1. We measured the mRNA transcription levels of four genes of down-regulated proteins (HisB, PGM, leuB and IDH).

As shown in Fig. 1. Four genes are all down-regulated .To sum up, all the results by RT-PCR analysis were consistent with the iTRAQ analysis.

Table 1 The primers used for real-time PCR in the experiment.

name Sequence (5′–3′)

HisB-F TACTTCTGTATCACCATT

HisB-R ACTATCTATCTCACTTGC

PGM-F TGACATCCATACCACAAA

PGM-R CAAAACCAATATCCAGAG

leuB-F GCACAAGCATCAACAAGT

leuB-R AAAAACGCCATAAGAAAT

IDH-F CACGAACTAAACGCTCAG

IDH-R AAGAAAATCGAATGGAAA

16sRNA-F CGGGCAATTTGTTTAGCA

16sRNA-R ATTAGGTGGAGCAGGTCA


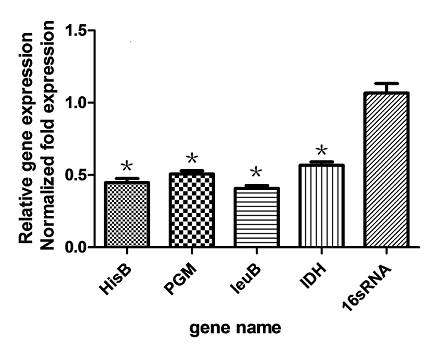


Fig. 1 Effect of 1/2MIC of aspirin on mRNA decreased expression of genes in *Staphylococcus xylosus* ATCC700404. Data are expressed as means ± standard deviations. The expression was normalized to 16S rRNA. Controls refer to the absence of aspirin. Significantly different (*****p < 0.05) compared to untreated control bacteria.

Yang, Y.-B., Wang, S., Wang, C., Huang, Q.-Y., Bai, J.-W., Chen, J.-Q., et al. (2015a). Emodin affects biofilm formation and expression of virulence factors in Streptococcus suis ATCC700794. *Archives of Microbiology* 197(10)**,** 1173-1180. doi: 10.1007/s00203-015-1158-4.
